# Changes in online public opinions associated with various three-child supportive policies in China: Observational study using social media data over time

**DOI:** 10.1371/journal.pone.0306698

**Published:** 2024-07-24

**Authors:** Lijuan Peng, Tinggui Chen, Jianjun Yang, Guodong Cong

**Affiliations:** 1 School of Business Administration, Zhejiang Gongshang University, Hangzhou, China; 2 School of Artificial Intelligence and Electronic Commerce, Zhejiang Gongshang University Hangzhou College of Commerce, Hangzhou, China; 3 Department of Computer Science and Information Systems, University of North Georgia, Oakwood, Georgia, United States of America; Zhejiang University, CHINA

## Abstract

**Background:**

According to the Seventh National Census, China’s fertility rate is less than 1.5, marking a significant national issue with potential risks. To counter this low birth rate, the Chinese government has relaxed family planning policies and introduced supportive measures.

**Purpose:**

Changes in birth policy have attracted considerable attention from the people of China. This article aims to study the public’s response to the three-child support policy using Weibo as a window. The goal is to provide a more balanced evaluation of current perspectives, enabling policymakers to formulate better fertility information, particularly when anticipating a poor public response to controversial policies.

**Methodology:**

This research uses a crawler to gather data from Sina Weibo. Through opinion mining of Weibo posts on the three-child policy, Weibo users’ online opinions on the three-child policy are analyzed from two perspectives: their attention content and sentiment tendency. Using an interrupted time series, it examines changes in online views on the policy, matching policy documents to the time nodes of Weibo posts.

**Findings:**

The public has shown great interest in and provided short-term positive feedback on policies related to improving maternity insurance, birth rewards, and housing subsidies. In contrast, there has been a continuous negative response to policies such as extending maternity leave, which has particularly sparked concerns among women regarding future employment and marital rights protection. On social media, the public’s attention to the three-child birth policy has focused mainly on the protection of women’s rights, especially legal rights after childbirth, and issues related to physical and mental health. Child-rearing support and economic pressure are also hot topics, involving the daily expenses of multichild families, childcare services, and housing pressure. However, this study also revealed that infertile or single women express a strong desire to have children, but due to limitations in the personal medical insurance system, this desire has not been fully satisfied.

**Contributions:**

Our study demonstrates the feasibility of a rapid and flexible method for evaluating the public response to various three-child supportive policies in China using near real-time social media data. This information can help policy makers anticipate public responses to future pandemic three-child policies and ensure that adequate resources are dedicated to addressing increases in negative sentiment and levels of disagreement in the face of scientifically informed but controversial, restrictions.

## Introduction

According to the data from China’s Seventh National Census, the number of new births in China was 12 million in 2020, decreasing by 18% from 2019 to 33% from 2016, when the two-child policy was initially released. In 2020, China’s total fertility rate was 1.3, lower than the global average of 2.45 [1]. To solve the current population problem, the “separate two-child” policy was officially implemented in December 2013, and the “universal two-child” policy was officially implemented in January 2016. However, after the implementation of the relevant policies, China’s low birth rate has not changed, and the persistently low population fertility rate has further raised concerns about China’s “negative population growth” [2]. Given that the cost of childbirth in China is relatively greater than that of income in most developed countries and is similar to that of South Korea, which has one of the lowest birth rates, China needs to more vigorously encourage childbirth than these other countries to increase its birth rate to the level of developed countries. The Political Bureau of the CPC Central Committee implemented the policy that a couple can have three children and issued a series of three-child supportive policies to improve the three-child policy, which is another major reform after the universal two-child policy [3]. Indeed, the three-child policy cannot be addressed by a single policy alone. This requires the backing of equally significant supporting policies [4]. This article primarily discusses these supporting measures for the three-child policy. At present, maintaining an appropriate fertility level is an important task, and fertility has become an important issue of concern to the nation and the public. The transition from the two-child policy to the three-child policy has sparked extensive discussions among the public, with mainstream media playing an authoritative and influential role. This is achieved through the continuous gathering of public opinion and the presentation of expert views, which helps shape a certain momentum of public opinion in favor of these policies. This momentum largely guides the public’s agenda and discussion topics. In particular, mainstream media has heightened awareness among women regarding the risks associated with childbirth, playing a dual role in both amplifying and mitigating the impact of these risks on the desire to have children [5]. In the realm of social media, women’s heightened stress responses to childbirth risks further contribute to the propagation of ‘fear of childbirth’ behaviors. This collective risk-avoidance emotional expression leads to a degree of over precaution against childbirth risks [6].

According to agenda-setting theory, it is well established that regulating public opinion has become an important aspect of social governance, playing a key role in the formulation, implementation, and evaluation of government policies [7]. The change in news reporting methods has influenced the public’s perception of current important issues. With the development of the internet, the power of traditional media has gradually shifted to the internet, and the media agenda has consequently transitioned to align more closely with the public agenda. This shift has empowered citizens to promptly access the government’s latest information through social media channels while also enabling authorities to gain deeper insights into public sentiments, concerns, and demands through user-generated content. Consequently, leveraging big data from social media platforms or public opinion insights derived from online forums has emerged as a crucial method for gauging public preferences and addressing practical issues. However, without proper regulation and oversight by the government, the amalgamation of public opinion, media coverage, and online expert analysis online can potentially magnify and disseminate negative sentiments within communities, thereby impacting the public’s reception of policies. Therefore, for policymakers and researchers, utilizing internet platforms to understand the attitudes of the Chinese public toward policies, as well as their responses and feedback to policy changes, is highly important. In the field of fertility policy, leveraging big data to analyze public sentiment and support on social media helps policymakers gain a more comprehensive understanding of public opinions and needs. This can provide valuable insights for predicting the future development trends of China’s fertility policy, further enhancing the top-level design of population policies [8]. Furthermore, these research findings represent a significant advancement in the in-depth exploration of agenda-setting theory and policy impact, offering novel ideas and methodologies to propel the field of policy development forward. This study, grounded in agenda-setting theory, examines the “three-child supportive policy” through the lens of public discourse. It analyses public opinion on policy in the context of social realities and policy changes, exploring the potential for public opinion regulation on fertility issues. The key questions include the following: What are the hot topics generated by the public on the internet in response to policy adjustments? In other words, what is the focus of the public agenda, and what aspects does it cover? How is the correlation between public discourse and policy established? How does the public respond to policy agendas? How does public opinion evolve in response to policy agendas? Specifically, how do various types of policies influence the public’s positive and negative attitudes, both immediately and over time? What are the specific manifestations of changes in people’s emotional inclinations?

Most of the existing research on fertility policy has combined with medicine [9], finance [10], and other disciplines to explore the impact of policy implementation on various fields, and its sociology has focused mainly on fertility policy [11], problems [12], and institutional responses [13] through traditional statistical data. Demography measures the fertility willingness of a certain population group [14], focusing on fertility macro trends [15] and fertility prediction [16]. Journalism and communication research has focused more on the external dissemination of China’s fertility policy [17]. Research on fertility concepts and attitudes mainly relies on questionnaire surveys and field interviews [18,19]. Although survey interviews are widely recognized as a fundamental and effective method of data collection in the social sciences, existing academic literature has revealed significant limitations in studying fertility perspectives [20]. One major challenge is the difficulty of accurately quantifying highly subjective and abstract concepts such as perceptions and attitudes through questionnaires [21]. Additionally, it can be challenging to measure an individual’s full understanding of the nuances and implications of a question through simple scoring [22]. Furthermore, fertility concepts are particularly prone to change and are heavily influenced by social and cultural factors [23]. For instance, the global average fertility rate has decreased from approximately 5 children per woman in the 1960s to 2.4 children per woman in 2021, highlighting the rapid evolution of fertility perceptions. Questionnaire surveys often lack timeliness, making it difficult to track these changes promptly or reflecting the uncertainty of fertility attitudes in an era of frequent fertility policy changes and rapidly shifting attitudes [24,25].

In addition, compared with traditional data sample sources, China’s online discussion platforms are an important source for understanding the dynamics of public opinion on specific issues and policies [26], and social media not only provides a rich source of data but also provides a cheap and efficient way to detect and evaluate such information. Big data has been suggested to mitigate the limitations of traditional questionnaire surveys and field interviews [27]. First, big data offers high timeliness and cost-effectiveness, enabling it to reflect real-time developments, thus reducing the delay often associated with questionnaire surveys. Second, it addresses the challenge of measuring abstract features that may be challenging to capture through traditional surveys. The language and immediate emotional responses of individuals on social media platforms indirectly convey such abstract information, which is often difficult to capture through traditional survey methods. Therefore, social media, particularly platforms such as Weibo, have emerged as widely adopted channels for citizens to communicate, discuss issues, and express opinions. Social media platforms enhance government openness and transparency, promote public oversight of government actions, and serve as crucial channels for government policy dissemination. They are also important platforms for scholars to explore information related to government-public interactions [28,29].

In recent years, scholars have utilized data from the Weibo platform to investigate online public sentiment following the release of fertility policies. For instance, Wang and Song analyzed the opinions of Chinese netizens on Weibo regarding the single-child policy and the comprehensive two-child policy. They assessed the changes in the attitudes of Weibo users toward the two-child policy in terms of attention intensity and emotional tendencies, corroborating their findings with national population data from the National Bureau of Statistics for the years 2011 to 2016 [30]. Han et al. conducted content analysis on Weibo posts related to China’s fertility policy over the past four years, examining discourse expression, topic changes, and public mentality [31]. Liu et al. performed critical discourse analysis on public posts after the abolition of the one-child policy, identifying dominant discourse responses and implications for prenatal care [32]. Through text analysis, Li et al. utilized web crawlers to assess public evaluations of two-child fertility-related issues on the internet and discerned attitudes through text analysis [33]. Lu et al. employed Weibo topic modeling for topic analysis on second-child policy-related blog posts to understand user concerns [34]. Ma et al. investigated the information transmission mechanism of the Weibo platform and determined public concerns through the retrieval of second-child policy-related keywords [35]. Gao conducted data mining on social platforms, finding a decade-long negative fertility sentiment with a positive impact from the two-child policy [36]. Shi and Cui explored the spatiotemporal evolution and influencing factors of network attention to fertility issues, discovering a significant positive correlation between fertility network attention and the fertility rate, reflecting fertility rate changes to some extent [37].

Existing research on fertility policies primarily focuses on the two-child policy, with scant attention devoted to matters pertaining to the recently implemented three-child policy on Weibo. While the academic community has conducted in-depth research on fertility policies, few studies based on social media have analyzed the online public sentiment toward the three-child support policy and explored the public’s views and demands on the policy. Specifically, from the perspective of public agenda setting, this study aims to extract the willingness of individuals to have three children and gauge the public’s attitudes and emotional tendencies following the policy’s release, by mining publicly posted network information from different regions on social media. Furthermore, as supported by the literature, the widespread adoption of Weibo in Chinese society has prompted individuals to employ this emerging social media platform to voice their perspectives and opinions on societal matters. Weibo serves as a new social media platform for researchers to gather and analyze public sentiments and emotions regarding fertility issues. Although discussions on fertility policy may not necessarily lead to collective action among netizens, Weibo’s online comment information provides valuable insights into Chinese netizens’ perspectives on the three-child support policy. Therefore, this article collects trending topics and blog posts related to the three-child support policy and constructs a semantic analysis model of policy topics from the perspectives of sociology and communication, which is of significant importance for the government’s comprehensive and multiperspective decision-making.

Building on existing research, this article uses data from Sina Weibo, a leading Chinese microblogging platform, to track public opinion shifts related to the three-child supportive policy. It analyzes public focus, demands, and emotional responses to policy. This approach aids in understanding public sentiment changes and adjusting future policy directions based on real needs. Therefore, this paper conducts an in-depth analysis of unstructured social media comments pertaining to the three-child supportive policy, utilizing agenda-setting theory as a foundation. This study employs emotion analysis and grounded theory to discern the thematic content and public emotional tendencies following the policy’s announcement, summarizing public focus and diverse perspectives on the policy. An interrupted time sequence model is constructed to examine the transient and enduring effects of various policy types on public sentiment, both positive and negative. This study further explores public cognition, emotional responses, and reactions during policy implementation. The ultimate aim is to provide recommendations for future government releases of related three-child supportive policies.

## Method

**Fig 1:**
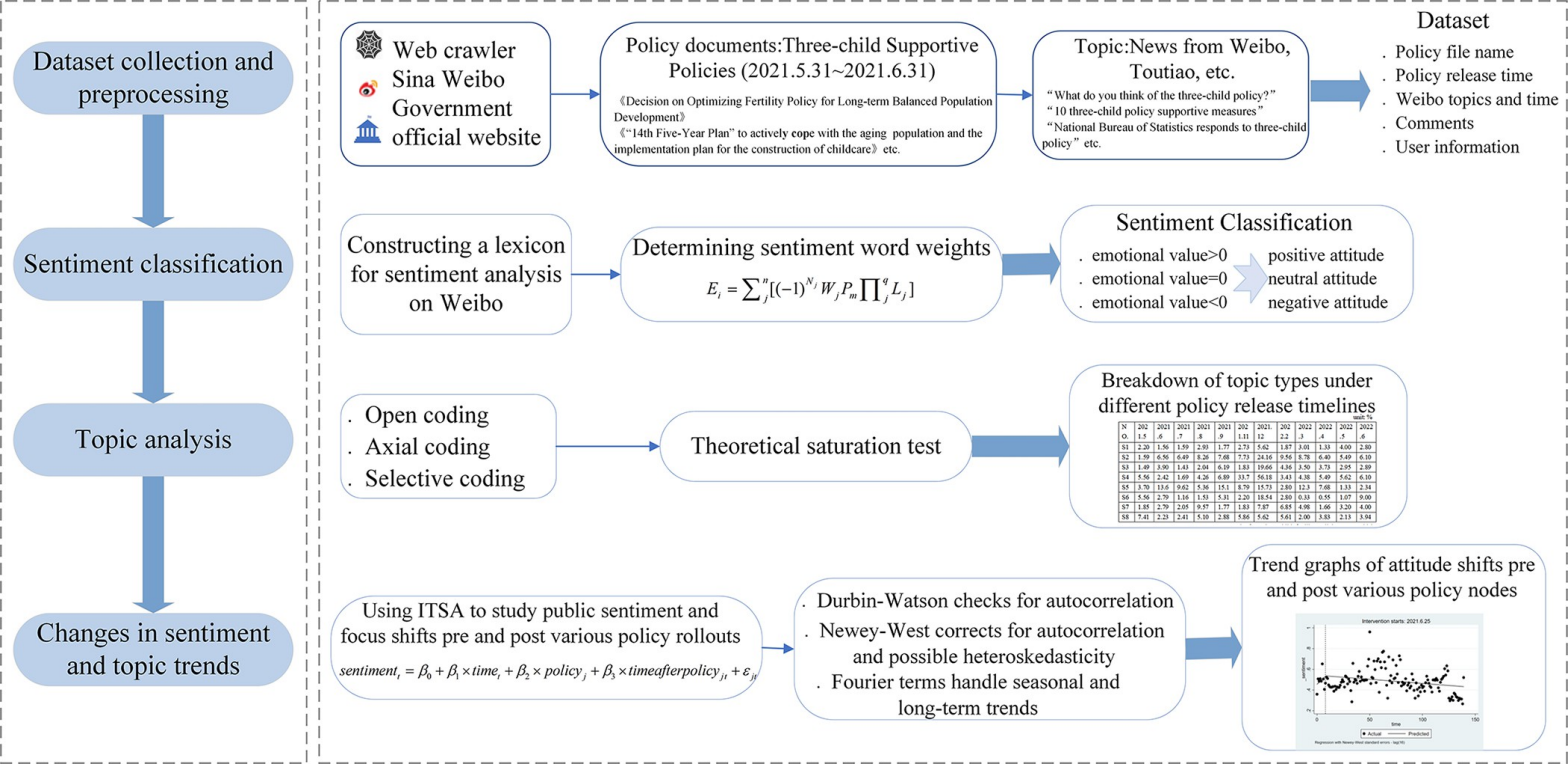
Research framework.

This study uses web scraping to gather data and text mining to process unstructured data. The research framework (Fig 1) and ideas are as follows:

Text data related to policies are scraped and quantified through text mining techniques, and statistical methods are ultimately utilized to quantify policy effects.

Data collection and preprocessing: Data collection primarily involves two aspects of the agenda-setting theory: the policy agenda and the public agenda. First, an algorithm is utilized to automatically crawl the database of three-child support policies from the websites of 31 provincial and municipal governments during the period from May 31, 2021, to June 31, 2022. This database includes the location, date, and department name of each policy document. Second, policy documents from the same timeframe were manually searched on two professional policy websites, pkulaw.cn and lawnew.cnki.net, to establish a database of the three-child support policy promulgated by the central government and various provinces and cities. These collected policy documents encompass laws, regulations, and ordinances issued by government departments at the central, provincial, and prefecture levels. Subsequently, the manually collected file information is compared with the information obtained by the automatic web crawler to determine the policy text corpus for the study (refer to Table 1 below). The policy text corpus is then cleaned to remove null values and stop words. Next, the term frequency-inverse document frequency (TF-IDF) method is employed to identify high-frequency keywords, followed by manual screening and filtering. Common words that are not clearly related to the analysis of policy features, such as ‘three-child birth’, and words that do not significantly impact policy feature analysis, are removed, thereby sorting out the effective high-frequency words necessary for mining topics related to the policy. Based on the high-frequency keywords obtained above, word2vec technology is utilized to expand related content and supplement the key content of the subsequently crawled blog posts. Additionally, considering the professionalism and rigor of policy text content and the existence of some low-frequency but important words, we supplement some low-frequency keywords and finally obtain the mining dictionary of the three-child support policy text.

**Table 1:** Topics related to the three-child policy in media reports.

No.	Time	Topic
**C1**	2021.5.31	What do you think of the three-child policy?
		What changes will the three-child policy bring?
		What is your opinion of the three-child policy?
**C2**	2021.6.1	10 three-child policy supportive measures
		Delegates propose extending maternity leave to 3 years
**C3**	2021.6.16	National Bureau of Statistics responds to three-child policy
**C4**	2021.6.25	Yueyang Bureau of Statistics recommends the introduction of a three-child incentive policy (to encourage natural births, shorten the birth cycle of the second and third children, and recommend the cancellation of extracurricular and online tutoring classes)
**C5**	2021.7.20	The three-child supportive policies are released (abolish social maintenance fees, fully decouple household enrollment and entry levels from personal fertility, support the pilot program of paternal leave in places where conditions permit, and encourage and support conditional kindergartens to enroll children aged 2 to 3, etc.)
		The woman who gives birth to three children enjoys 98 days of national statutory maternity leave
		Fully decouple household enrollment and entry levels from personal fertility
		Three-child policy supporting measures announced
**C6**	2021.7.26	State Medical Insurance Bureau requires timely payment of three-child birth allowance
		The three-child cost is included in the payment scope of maternity insurance
		The three-child cost of insured female employees is included in the guarantee
**C7**	2021.7.28	Panzhihua government gives a monthly subsidy of 500 yuan per child for family with two or three children
**C8**	2021.8.5	30 days of birth leave after giving birth to three children after May 31 in Beijing
		Women enjoy 30 days of birth reward for giving birth to three children in Beijing
		Beijing Health Commission responds to the three-child incentive policy
**C9**	2021.8.8	Is it effective to encourage three children by maternity subsidies?
**C10**	2021.8.20	Three-child birth policy officially entered into law
		Population and Family Planning Law is revised
		Law guarantees the implementation of the three-child birth policy and supportive measures
**C11**	2021.9.13	Women with three children registered in Guangdong can enjoy 80 days of bonus leave
**C12**	2021.11.24	Beijing prioritizes allocation of public rental housing for families with two or more children
**C13**	2021.12.24	Fully implement online birth registration
**C14**	2022.2.18	Anhui promotes the implementation of the three-child birth policy
**C15**	2022.3.23	Many places clearly put forward the childcare subsidy system
**C16**	2022.4.26	Families with two and above children can buy another suite in Wuxi
**C17**	2022.4.27	Wuhan government allows families have three children who can study in the same public school
**C18**	2022.5.6	Guangdong will no longer need approval to new birth in May
**C19**	2022.5.7	Henan implements interprovincial registration of three-child births
**C20**	2022.5.11	Raise the standard of special assistance for family planning families since July 1
**C21**	2022.5.14	Nanjing families with two children and above can purchase an additional set of commercial housing
**C22**	2022.5.30	Chengdu distributes tens of millions of childcare consumption subsidies
**C24**	2022.6.24	Shaanxi Hanzhong government gives 10,000 yuan for one new birth

Additionally, a dictionary is constructed based on the key content and keywords extracted from the aforementioned policy documents. Subsequently, this dictionary is utilized to search for the main topics discussed in media reports on mainstream social media platforms such as Weibo, Toutiao, and The Paper (refer to Table 2 below). Through this method, the aim is to explore the correlation between the public agenda and the policy agenda. These platforms boast a large user base, provide stable and reliable news information, and wield considerable influence in the social media industry. Therefore, the decision was made to collect netizens’ opinions on these platforms. To align with the policy documents, relevant news articles published on social media platforms are collected at each time point when the policy documents are released. The netizens’ comments on these articles are then crawled, serving as the primary data source for the research. For the public agenda, Python is employed to crawl the netizens’ comment data on the corresponding topics from May 31, 2021, to June 31, 2022. The data were preprocessed by removing duplicate comments, repetitive expressions, shorter sentences, and unclear or meaningless sentences. If the proportion of meaningless comments in the final sample exceeds 20%, those comments are excluded. Finally, the total number of comments for each topic during this period is tabulated. These comments are not only generated immediately after media reports on policy information but also continue to accumulate and evolve throughout the entire period. Therefore, the statistical data presented in this article reflect netizens’ ongoing attention and discussion on various topics. Materials were gathered from the Chinese Weibo website (akin to YouTube), accessible at https://weibo.com/. All the data used in this research adheres to the relevant terms and conditions of the data sources. Necessary permissions and approvals are obtained to collect and analyze these data, ensuring full compliance with the provisions of the data use agreement and privacy policy.

**Table 2:** Number of comments.

NO.	2021.5	2021.6	2021.7	2021.8	2021.9	2021.11	2021.12	2022.2	2022.3	2022.4	2022.5	2022.6
**C1**	554	125	-	340	-	-	-	300	293	-	-	-
**C2**	-	296	140	200	-	-	-	-	-	-	-	-
**C3**	-	370	-	-	-	-	-	-	-	-	-	-
**C4**	-	247	-	-	-	-	-	-	-	-	-	-
**C5**	-	-	500	-	-	-	-	-	-	-	-	-
**C6**	-	-	666	-	-	-	-	-	-	-	-	-
**C7**	-	-	287	-	-	-	-	-	-	-	-	-
**C8**	-	-	-	298	339	546	-	-	-	-	-	-
**C9**	-	-	-	229	-	-	455	290				
**C10**	-	-	-	115	-	-	-	-	-	-	-	-
**C11**	-	-	-	-	274	-	-	-	-	-	-	-
**C12**	-	-	-	-	-	627	-	-	-	-	-	-
**C13**	-	-	-	-	-	-	639	-	-	-	-	-
**C14**	-	-	-	-	-	-	-	282	-	-	-	-
**C15**	-	-	-	-	-	-	-	-	480	-	-	-
**C16**	-	-	-	-	-	-	-	-	-	354	-	-
**C17**	-	-	-	-	-	-	-	-	-	179	-	-
**C18**	-	-	-	-	-	-	-	-	-	-	281	-
**C19**	-	-	-	-	-	-	-	-	-	-	130	-
**C20**	-	-	-	-	-	-	-	-	-	-	124	-
**C21**	-	-	-	-	-	-	-	-	-	-	120	-
**C22**	-	-	-	-	-	-	-	-	-	-	120	-
**C24**	-	-	-	-	-	-	-	-	-	-	-	350

Sentiment Intensity Calculation: Quantify the sentiment intensity values of netizen comments at the time points of policy document release (from May 31, 2021, to June 31, 2022). A sentiment weight calculation method is introduced that combines the comment text with existing negation words in the Chinese grammar dictionary, HowNet dictionary, and common Weibo language. Based on the sentiment score, netizen comment attitudes are categorized as positive (sentiment score equals 0), neutral (sentiment score greater than 0), or negative (sentiment score less than 0). Additionally, the sentiment scores are normalized to ensure that the comment attitude values for each day fall within the range of [0, 1].

Topic Cluster Analysis: Drawing on grounded theory, this study aims to discern the public’s focal points and demands from comments following the release of the three-child support policy, thereby pinpointing the primary concerns on the public agenda. This paper endeavors to comprehend netizens’ viewpoints, emotions, and behavioral motivations through the analysis of comment data. Grounded theory provides detailed and rich descriptions, whereas machine learning clustering algorithms primarily uncover patterns and trends in data. Therefore, grounded theory offers more advantages than clustering algorithms. The process primarily involves four steps: open coding, axial coding, selective coding, and theoretical saturation testing. Initially, open coding utilizes Nvivo11 software to categorize online comment data, resulting in the identification of initial concepts. Subsequently, the initial concepts are compared and analyzed, with those appearing fewer than five times being eliminated. Based on semantic and logical relationships, the initial concepts are then organized into 21 initial categories. Next, categories are classified based on their mutual relationships and logical order, resulting in four main categories. The core category is then identified from these main categories, and the connection relationships between the core category, main categories, and other categories are analyzed. Behavioral phenomena and contextual conditions are depicted in the form of a ‘storyline’. Upon completion of the ‘storyline’, a new substantive theoretical framework is developed. Finally, a theoretical saturation test is conducted on 1000 reserved comment data points. If no new initial concepts, categories, or structural relationships emerged during coding, the theoretical saturation test was considered successful.

Public Sentiment and Topic Trend Changes: Interrupted time series analysis is utilized to quantitatively examine changes in the public’s emotional tendencies and focal points following the release of different policy information, building upon the policy topics highlighted in (3). Furthermore, variations in public attitude shifts across different policy types are investigated, aiming to elucidate the characteristics of public feedback on the policy agenda. This approach enables the generation of comparative charts of the public’s emotional tendencies before and after the dissemination of each policy type, facilitating a quantitative assessment of both immediate and sustained impacts.

### Data collection and preprocessing

At the national level, significant reform measures have been introduced, ranging from canceling social support fees and abolishing relevant penalty regulations to decoupling household registration, school enrollment, and employment from personal fertility status and revising the Population and Family Planning Law. At the local and departmental levels, various departments have promoted effective connections between relevant people-benefiting policies and childbirth policies to effectively address public concerns. This section examines relevant three-child supportive policies at the national and local levels, as shown in Table 1.

With the advancement of technology, the media landscape is undergoing significant transformations, leading to a shift in the influence dynamics of traditional media. According to agenda-setting theory’s concept of network agenda setting, the emergence of the internet has further empowered the public’s role in shaping the agenda. Therefore, this study utilizes the public agenda framework from agenda-setting theory to conduct an in-depth analysis of public sentiment following the adjustment of the three-child policy. Following the introduction of the three-child supportive policy by the government, major media outlets reported it online, leading to a series of public comments on online platforms. These comments contain valuable information on public opinion and attitudes toward the policy, the topics of focus postpolicy release, factors influencing people’s desire to have children, and fertility issues people most want improved. This study, therefore, searches for related topics reported by media on platforms such as Weibo based on key content and keywords in the policy documents in Table 1 before and after the policy releases. Python is used to crawl user comment data corresponding to these topics during this period. The results are shown in Table 2.

Invalid and erroneous data, which may occur during the crawling process and could skew the results of comment data analysis, need to be removed. After data preprocessing, all comments on each topic between 2021.5.31 and 2022.6.31 were tallied. These comments were not only generated immediately after the policy information was reported by the media but also continued to be generated and developed throughout the entire period. Thus, the statistics in this study reflect the sustained attention and discussion of various topics by netizens, as shown in Table 3.

**Table 3:** Samples of three-child supportive policies.

No.	Name	Time	Department	Region/purpose
**1**	Decision on Optimizing Fertility Policy for Long-term Balanced Population Development	2021.5.31	Meeting of the Political Bureau of the CPC Central Committee	National/Three-child policy launched
**2**	Notice on the issuance of the “14th Five-Year Plan” to actively cope with the aging population and the implementation plan for the construction of childcare	2021.6.25	National Development and Reform Commission	National/Childcare subsidy
**3**	Decision of the Central Committee of the Communist Party of China and the State Council on Optimizing Fertility Policy for Long-term Balanced Population Development	2021.7.20	Central Committee of the Communist Party of China, State Council	National/Three-child policy launched
**4**	Notice on the Work of Maternity Insurance in Support of the Three-Child Policy by Office of the National Health Insurance Administration	2021.7.26	Office of the National Healthcare Security Administration	National/Fertility insurance
**5**	Sixteen Policy Measures on Promoting Human Resources Gathering	2021.7.28	Office of the CPC Panzhihua Municipal Committee, Office of the Panzhihua Municipal People’s Government	Panzhihua/Fertility subsidy
**6**	Notice on Implementing the Decision of the CPC Central Committee and State Council on Optimizing Fertility Policy for Long-term Balanced Population Development	2021.7.29	National Health Commission	National/Three-child policy launched
**7**	Beijing Regulations on Population and Family Planning	2021.8.4	Health Commission	Beijing/Fertility leave and incentives
**8**	Notice on Strengthening the Management of Public Rental Housing Eligibility Review and Allocation	2021.8.18	Beijing Municipal Committee of Housing and Urban‒Rural Development	Beijing/Housing subsidy
**9**	Notice on the Work of Maternity Insurance to Support the Three-Child Policy	2021.8.19	Anhui Provincial Medical Insurance Bureau	Anhui/Fertility insurance
**10**	Review of the Law of the People’s Republic of China on Population and Family Planning (Amendment)	2021.8.20	Constitutional and Legal Committee of the National People’s Congress	National/Support
**11**	Notice of Guangdong Provincial Health Commission Transmitting the National Health Commission’s Implementation of the Decision on Optimizing Fertility Policy to Promote Long-term Balanced Population Development	2021.9.13	Guangdong Health Commission	Guangdong/Fertility leave
**12**	Guidance on Improving the Birth Registration System by the General Office of the National Health Commission	2021.12.9	General Office of the Health Commission	National/Birth registration
**13**	Implementation Plan on Optimizing Fertility Policy for Long-term Balanced Population Development	2022.3.1	General Office of Jiangsu Provincial Committee of the Communist Party of China	Jiangsu/Housing subsidy
**14**	Notice on Increasing the Support Standard of the Special Support System for Family Planning Families	2022.4.20	Ministry of Finance, Health Commission	National/Assistance system
**15**	Notice on Active Promotion of Improvement of Housing Conditions for Families Having Two or More Children	2022.4.25	Wuxi Municipal Health Commission	Wuxi/Housing subsidy
**16**	Notice on the Enrollment of New Students during Compulsory Education Period in 2022	2022.4.27	Wuhan Education Bureau	Wuhan/Education subsidy
**17**	Management of Birth Registration of Guangdong Health Commission	2022.5.1	Guangdong Provincial Health Commission	Guangdong/Birth registration
**18**	Implementation Plan for Optimizing Fertility Policy for Long-term Balanced Population Development in Henan Province	2022.5.7	General Office of Henan Government	Henan/Birth registration
**19**	Notice on Optimizing Fertility Policies for Long-term Balanced Population Development in Hanzhong	2022.6.23	Hanzhong Municipal Health Commission	Hanzhong/Fertility subsidy

### Sentiment analysis of the three-child supportive policies

In order to better understand the effects of each policy on public attitudes, this section will utilize a sentiment analysis method based on a sentiment lexicon. This will help to reveal the overall sentiment towards the three-child supporting policies both before and after their enactment. To ensure accuracy in the sentiment labeling process, we will use manual labeling. However, as labeling results may vary among individuals, multiple annotators will be invited to label the same dataset. In cases where there is inconsistency in labeling, the majority rule will be followed, and the labeling with the most support will be chosen as the final result.

#### Sentiment dictionary construction

This paper utilizes a sentiment analysis method based on a sentiment dictionary. The key to this method is constructing a comprehensive sentiment dictionary that effectively meets the needs of sentiment analysis. The earliest foreign sentiment dictionary, SentiWordNet, combines words with similar meanings from WordNet and assigns them positive or negative polarity scores, which accurately reflect the user’s emotional attitude. In contrast, the Chinese sentiment dictionary mainly consists of NTUSD, HowNet, and the Sentiment Vocabulary Ontology Library. These three types of sentiment dictionaries contain varying numbers of positive and negative words, and many sentiment analysis researchers have extensively studied and utilized them. However, with the constant emergence of new words in modern online society, the sentiment lexicon needs regular expansion.

In this paper, we have integrated the HowNet dictionary, the Synonym Forest from HIT, the authoritative Sentiment Vocabulary Ontology Library (DUTSO) compiled by Dalian University of Technology, and the NTUSD dictionary compiled by National Taiwan University to create a baseline sentiment lexicon. This lexicon is then combined with various types of semantic rules to determine the sentimental tendency of online comments. Additionally, we have included a negation lexicon and a degree adverb lexicon, considering the semantic characteristics of Chinese, as shown in Table 4.

**Table 4:** Selection of the degree adverb.

Degree	Emotional intensity	Degree adverb	Number
**large**	2	very, especially	47
**medium**	1.5	too, more	39
**small**	0.5	relatively, a little bit	31

In addition to the use of adverbs of degree, continuous punctuation is often employed when users are commenting (e.g., “!!!!!”, “????”) to reflect their emotions. In this regard, the end punctuation of comments will be recognized here, and the emotional intensity of various punctuations will be set, as shown in Table 5 below.

**Table 5:** Emotional intensity of punctuation.

Punctuation	Emotional intensity
**!×n(n≥1,n represents the number with same punctuation style)**	1.5×n
**?×n(n≥2)**	1.2×(n-1)
**~×n(n≥1)**	0.8×n

#### Rule definition

In the process of constructing a sentiment lexicon, simply extracting sentiment words is not enough to accurately reflect the sentiment of comments. Therefore, the semantic rules of the context must also be considered in order to improve the accuracy of sentiment analysis. We use a convolutional neural network model to efficiently extract phrase representations with varying contextual distances, allowing for a more precise determination of the polarity and intensity of sentiment words [38].

Additionally, common linguistic phenomena such as hyperbolic expressions, sarcasm, and metaphors in online comments can affect the calculation of sentiment scores. For example, a statement like “You’re a genius!” may actually have a negative affective tendency if used sarcastically. To address this, we draw on the literature [39,40] on the sentiment tendency and calculation of special languages and use hybrid neural network models to recognize and properly handle these types of languages. In this paper, we employ two main approaches: first, to detect semantic skew, we calculate the difference in sentiment between two semantic blocks using the sentiment tendency and score from the sentiment dictionary, as well as sentiment words and negatives in the sentence. Second, to overcome the limitations of the sentiment lexicon, particularly in recognizing the sentiment of new words, we introduce the Word2Vec model to balance this deficiency. We use cosine similarity as a feature in our analysis by calculating the cosine similarity between the word vectors of sentiment words in the two semantic chunks. If there is a significant semantic deviation before and after, with an angle greater than 90 degrees, we can accurately quantify special semantic emotions. By synthesizing the above methods, we are able to accurately calculate the initial sentiment score (*E*_*i*_) of the *i*th comment:

$$E_{i}=\sum_{j}^{n}\left[{\left(-1\right)}^{N_{j}}W_{j}P_{m}\prod_{j}^{q}L_{j}\right]$$ (1)

where *W*_*j*_ is the *j*^th^ emotional word in the comment. *L*_*j*_ is the emotional intensity of the degree adverb before the *j*^th^ emotional word. *N*_*j*_ is the number of negative words before the *j*^th^ emotional word. Pm is the emotional intensity of the comment ending punctuation. Q is the number of degree adverbs before the *j*^th^ emotion word. According to the emotional score, public Weibo comment attitudes can be divided into three categories: positive (emotional score greater than 0), neutral (emotional score equal to 0), and negative (emotional score less than 0). Since neutral comments often represent objective statements of information, this paper aims to measure the subjective views of individuals with negative and positive attitudes, so neutral comment information is not considered. In addition, to determine the comment attitude serial value of each day, the emotion score of the same day is averaged and standardized, so that the comment attitude value of each day is within the range of [0,1].

#### Model validation and adjustment

**Fig 2:**
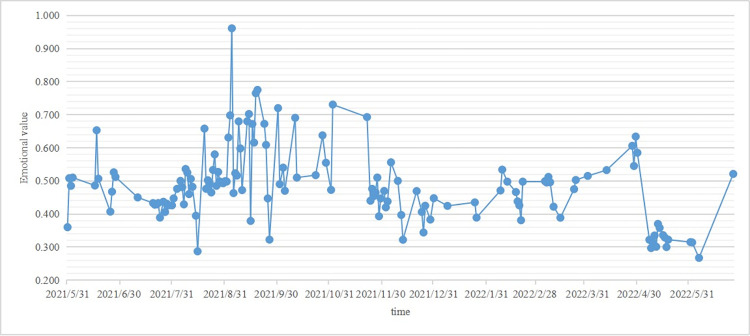
Comment attitude serial values from 2021.5 to 2022.6.

In sentiment analysis, there are different methods used, such as sentiment dictionaries and machine learning models. Despite their differences, metrics like accuracy and recall can still be used to validate and evaluate their effectiveness. These metrics are important in assessing the performance of sentiment analysis methods in real-world applications. Accuracy measures the percentage of correctly predicted sentiment categories, while recall evaluates the percentage of correct predictions among all comments that are actually for a certain sentiment. Both of these metrics reflect the performance of classification quality from different perspectives. In practice, higher accuracy and recall indicate more accurate and comprehensive classification results. In social media comment sentiment analysis, achieving 70%-85% accuracy and 60%–80% recall is generally considered a reasonable standard to aim for. The attitude sequence value are shown in Fig 2 below.

## Results

### Topic analysis of the three-child supportive policies

Based on empirical data, grounded theory summarizes the original data, i.e., from the bottom to the top, the data are continuously concentrated, conceptualized, categorized, and systematically explored, and then the theory is derived [41].

A coding group comprising four students was trained to analyze coverage. At the beginning of the evaluation, we trained the 4 raters in the method of “independent coding-proofreading-third-party opinion discussion” and then ensured that the internal reliability reached 0.9 or above before they began to evaluate the formal materials. All the formal comment assessments were completed according to the following steps:

Step 1: Initially, the coding team conducts training to define and unify the coding rules.

Step 2: One thousand sample comments are extracted, and each rater is asked to code these comments independently. We compared the coding results between the raters to obtain “interrater reliability” (interrater reliability = the number of entries with the same coding result/total number of entries, i.e., the consistency between all the raters’ coding results). The final results are unified according to the coding rules.

Step 3: If the interrater reliability is less than 0.9, we repeat the operations in Step 2 until it reaches 0.9. The coding team then begins to code formal samples.

Step 4: The raters read and code all the comments independently without interfering or discussing them with each other. Each comment can belong to one category or more.

Step 5: We compare the coding results of each rater to find comments that are inconsistent with the coding results.

Step 6: We establish a discussion group to reread all the comments and determine the coding results of comments that are inconsistent in Step 4. Finally, the coding team completed the coding of all the comments.

#### Open coding

This section uses Nvivo11 software to encode online comments to generate initial concepts, compares and analyzes the initial concepts, and combines the concepts of intersection, similarity, or overlap. Then, the initial concepts whose occurrence number is less than 5 times are removed. The initial concepts are categorized according to the semantic relationships and logical relationships between the initial concepts, and the initial concepts are summarized into 21 initial categories. Their open codes are shown in Table 6.

**Table 6:** Results of open codes.

Initial concept	Original data (part)
**Unmarried infertility, DINK**	It’s even better to be single and DINK Modern women are truly unwilling to give birth or marry
**Marriage costs (marriage leave, dowry)**	When my mother-in-law does not want the dowry, I will give birth First, implement the marriage leave. It is useless if the policy is not implemented. The marriage leave will be deducted 100 Yuan a day.
**Alleviate the loneliness of the only child**	I believe the three-child policy is good because I am the only child and I want a sister
**Social maintenance**	I thought the state paid for fertility, but actually, the state only cancelled fine. What’s the meaning of social maintenance fee?
**Infertility and single women’s childbirth costs included in medical insurance**	The state could include IVF infertility treatment in the full reimbursement of health insurance. Many people are anxious for IVF because they cannot give birth and it is too expensive if someone has the desire to give birth, so the state should strongly support. The state should include IVF infertility treatment in the full reimbursement of health insurance. Many people are anxious for IVF because they cannot give birth and it is too expensive if someone has the desire to give birth, so the state would be strongly support. Encouraging childbirth could be covered by health insurance
**Medical Insurance (Medical Insurance)**	Can we put medical expenses into health insurance? No health insurance or pension insurance without childbirth.
**Economic income pressure**	The pressure is caused by rising expenses except for wages. High house price and low wage
**Housing pressure (housing prices, allocation of public rental housing)**	Who dares to have a child without a house in Beijing? You have to buy at least five bedrooms to have three children. One for the couple, one for the parents who help with the baby, and three for the three children. They cannot live, let alone raise children.
**Gender discrimination in female employment (before pregnancy, during pregnancy and after pregnancy)**	Female workers are hard to seek employment. Job discrimination for female workers is worsened. Having a baby is the same as being unemployed, so how can you enjoy maternity leave?
**Protection of women’s rights and interests within marriage (equality between men and women, women’s human rights and legal protection, widowed marriage, etc.)**	Domestic violence problems are not solved, not alone having children. Implement marital women’s rights protection. Improve the legal protection of housewives.
**Maternity Insurance**	Can maternity insurance be more humane? Couldn’t maternity insurance for women who become pregnant after leaving a job be more practical and thorough in implementing the benefits already in place? People cannot enjoy fertility insurance if their social security does not exceed one year, which could be changed.
**Maternity allowance (government allowance for enterprises (corporate tax reduction) or individuals)**	Give additional tax breaks to companies with a high percentage of women in the business. Simplify the process from birth to subsidy application.
**Maternity leave (female maternity leave and male paternity leave)**	A year of maternity leave for the birth of a third child. I hope that the length of maternity leave for men and women can be adjusted to the same length. Therefore, women do not need to bring up children alone and do not worry about employment discrimination, which gives impetus to give birth. Consistent maternity leave time for men and women is the only way to fundamentally eliminate some employment discrimination.
**Fertility costs (pregnancy costs)**	It is unreasonable to pay all fees by yourself during pregnancy. The existing policy is not appealing. It may be effective if the state covers all medical fees during pregnancy.
**Fertility reward**	Reward for fertility is useful. It is difficult to have babies in the context of high house prices.
**Birth registration (online application, birth certificate)**	Birth registration can be done online. When ID cards for children can be done directly off-site? Go to birth control office frequently for registering the birth.
**Fertility fears (birth fears, postpartum depression, low self-esteem)**	Many women are now suffering from postpartum depression after giving birth. Pregnancy and childbirth is too painful I have never experienced in my life.
**Cost of living (daily living expenses)**	The state could cover all fees including milk powder fee, medical fee, tuition fee. It is ridiculous to pay 500 Yuan per month for one child.
**Parenting time (childcare services)**	Run more nurseries from 4 months to 4 years old to truly reduce the burden on mothers, who worry about parenting and can only choose to be full-time mothers. The main thing about having a baby is that there is no one to take care of the baby.
**Education costs (kindergarten tuition, extension of compulsory education, extracurricular make-up fees, tuition and miscellaneous fees, childcare fees)**	Twelve years of compulsory schooling could be promoted. First to solve the kindergarten problem. Three-year universal kindergarten is very expensive. It is difficult to spend 30000 Yuan a year on tuition. In addition to maternity leave, the state can involve kindergarten into compulsory education. At present, family spend 5000 Yuan and 7000 Yuan (early care included) per semester for one child. It is difficult to afford for three children.
**Education resource allocation (household registration, entrance qualification)**	Nine years of compulsory schooling in Beijing requires a Beijing household registration. Neighborhood kindergartens are either military or institutional kindergartens, and ordinary people can’t even get into a public or inclusive kindergarten.

#### Axial coding

The task of axial coding (associative logging) is to discover the potential logical connections between categories and to develop the main categories and their subcategories. In this paper, the different categories are classified according to their interrelationship and logical order at the conceptual level, and a total of four main categories are summarized. Each main category and its corresponding open coding categories are shown in Table 7.

**Table 7:** The main category formed by axial coding.

No.	Range	Concept
**S1**	Personal concept of marriage and childbirth	Unmarried infertility or DINK
		Alleviate the loneliness of the only child
**S2**	Constraints	Social maintenance fee
		Birth registration (online)
**S3**	Fertility medical insurance (insurance, social security, etc.)	Infertility or single woman childbirth costs included in health insurance
		Medical insurance
		Fertility Insurance (Fertility Insurance)
		Fertility costs
**S4**	Economic pressure	Economic income pressure
		Housing pressure (housing prices, public housing allocation)
		Cost of marriage (wedding leave, dowry)
**S5**	Women’s Coverage	Female employment gender discrimination (before, during and after pregnancy)
		Protection of women’s rights and interests within marriage (gender equality, women’s human rights and legal protection)
		Fear of childbirth (fear of childbirth, postpartum depression, low self-esteem)
		Maternity leave (female maternity leave and male paternity leave)
**S6**	Fertility benefits	Fertility benefits (government benefits for businesses (corporate tax deductions) or individuals)
		Fertility incentives
**S7**	Parenting security	Living cost of childbirth (daily living expenses)
		Parenting time (childcare services)
**S8**	Education security	Education costs (kindergarten tuition, extension of compulsory education, extracurricular fees, tuition and miscellaneous fees, childcare fees)
		Education resource allocation (household registration, entrance qualification)

#### Selective coding

The typical relationship structure of the main categories in the text is shown in Table 8.

**Table 8:** Process of selective coding.

Typical relationship structure	Range
**Family factors**	Personal concept of marriage and childbirth
	Female guarantee
**Economic factors**	Economic pressure
	Fertility benefits
**Social factors**	Childbirth security
	Education security
	Constraints
	Maternity and medical coverage

#### Theoretical saturation test

The theoretical saturation test completely tests the theoretical system formed by three coding processes of grounded theory. Here, the saturation test of the theory is conducted on the data of 1000 reviews set aside, and the results of the theoretical test revealed that there are no new initial concepts, new categories, or structural relations that emerged from the coding process.

#### Percentage of coding categories based on time sequence

Based on the coded classification of the online comments in Table 7, the percentage of people’s attention to each category of topics at different times is analyzed, and Table 9 shows the percentage of coding categories from 2021.5–2022.6.

**Table 9:** Percentage of coding categories.

NO.	2021.5	2021.6	2021.7	2021.8	2021.9	2021.11	2021.12	2022.2	2022.3	2022.4	2022.5	2022.6
**S1**	2.20%	1.56%	1.59%	2.93%	1.77%	2.73%	5.62%	1.87%	3.01%	1.33%	4.00%	2.80%
**S2**	1.59%	6.56%	6.49%	8.26%	7.68%	7.73%	24.16%	9.56%	8.78%	6.40%	5.49%	6.10%
**S3**	1.49%	3.90%	1.43%	2.04%	6.19%	1.83%	19.66%	4.36%	3.50%	3.73%	2.95%	2.89%
**S4**	5.56%	2.42%	1.69%	4.26%	6.89%	33.70%	56.18%	3.43%	4.38%	5.49%	5.62%	6.10%
**S5**	3.70%	13.5%	9.62%	5.36%	15.0%	8.79%	15.73%	2.80%	12.2%	7.68%	1.33%	2.34%
**S6**	5.56%	2.79%	1.16%	1.53%	5.31%	2.20%	18.54%	2.80%	0.33%	0.55%	1.07%	9.00%
**S7**	1.85%	2.79%	2.05%	9.57%	1.77%	1.83%	7.87%	6.85%	4.98%	1.66%	3.20%	4.00%
**S8**	7.41%	2.23%	2.41%	5.10%	2.88%	5.86%	5.62%	5.61%	2.00%	3.83%	2.13%	3.94%

From May 2021 to June 2022, a total of 19 three-child supportive policies were widely publicized on media platforms, including national or local policies, and 24 derived hot topics were generated. By mining online comments on the hot topics, this paper summarizes the hot spots of public concern about related supportive policies, such as the personal concept of marriage and childbirth, maternity medical protection, women’s protection, and economic pressure, etc. The public agenda focuses on women’s protection in several aspects: first, addressing women’s fear of childbirth through medical means and disseminating relevant childbirth knowledge among those who have not given birth to alleviate such fear; second, enhancing legal rights protection for women in both the workplace and family post childbirth to mitigate gender inequality resulting from women’s childbirth. Drawing on the experiences of Europe and other countries, integrating the concept of gender equality in family roles into the parental leave system and legally stipulating that fathers share family responsibilities can not only promote equal opportunities for men and women in the workplace and shared responsibilities in household chores but also help eliminate gender discrimination in the job market. Third, more attention and assistance are needed for the physical and mental health of women after childbirth, including addressing postpartum depression, feelings of inadequacy, and other mental health issues. According to the 2022 “China Urban Women’s Childbirth Report,” childbirth often entails physiological pain and emotional challenges, which aligns with our research findings. In addition to women’s protection, the public also emphasizes child-rearing protection and economic pressure. Child-rearing protection encompasses managing the daily expenses of raising multiple children and developing childcare services, while economic pressure mainly arises from housing expenses after having multiple children. According to a questionnaire survey conducted by the People’s Think Tank on various genders, more than 80% of respondents cited high income pressure, rising education costs, and a challenging employment environment as the primary reasons for reluctance to bear children. These survey results are consistent with our research findings, indicating the representative nature of the data collected in this article.

From the percentages of topics in different years, it can be observed from a macro perspective that as fertility policies continue to evolve, public agendas increasingly reflect actual concerns. The public’s attention to the ‘child-rearing guarantee’, ‘women’s protection’, and ‘education guarantee’ for multiple children has remained consistent from May 2021 to June 2022. However, the focus on ‘institutional measures’, ‘birth medical insurance’, and birth subsidies’ significantly increased only in December 2021. Comments regarding ‘birth medical insurance’ often mentioned the fertility desires of infertile or single women. These groups express a strong desire for children but face challenges due to their personal circumstances or limitations within the medical insurance system. Many countries worldwide have implemented measures to safeguard the rights and interests of unmarried mothers and nonmarital children. For instance, the French government provides equal family subsidies to encourage childbirth, irrespective of marital status, with subsidies increasing based on the number of children. Regarding inheritance, the French Civil Code ensures equal rights for children born in and out of wedlock. The marriage rate among women in Nordic countries is lower than that in Japan, yet the fertility rate is higher. This is largely due to the tolerance of nonmarital childbirth in these nations, coupled with generous government support for child-rearing, enabling many single women to independently choose to have and raise children. Hence, China can draw valuable lessons from the progressive approaches adopted by these countries in implementing its policies. From the percentages of different topics in the same year, we can see that after the first release of the “Decision on Optimizing Fertility Policy to Promote Long-term Balanced Population Development” policy, people mostly held a negative attitude toward this policy, and the discussion on the policy focused mainly on the education security of children, especially the extra education expenses brought about by multiple children, such as kindergarten tuition, school fees, and extracurricular expenses. In addition, the public is concerned about the economic pressure of having three children and the employment discrimination women may face in all pregnancy processes. With the introduction of the three-child supportive policy and the release of the “14th Five-Year Plan for the Implementation of the Project of Actively Coping with Population Aging and the Construction of Child Care”, the public’s focus shifted to gender discrimination in women’s employment, and the construction of child care mentioned in the policy did not receive timely attention and feedback from the public.

Since then, the National Health Insurance Administration has proposed a notice to support maternity insurance for the three-child supportive policy and further optimize the three-child maternity policy [42]. However, the public’s attention is not concentrated on the discussion of maternity insurance but rather on the issue of how to balance maternity leave for women and accompanying maternity leave for men. The balance of maternity leave between men and women can directly lead to employment discrimination against women and the lack of protection of women’s rights within marriage [43]. Although the length of maternity leave for women has increased extended, women face both financial and postpartum mental stress during pregnancy, and whether women’s financial, employment, and marital rights will be legally protected during and after pregnancy is still the main concern of most women [44]. In terms of local policies, Beijing has made corresponding adjustments to its population and family planning regulations, mainly concerning incentives for three-child fertility and the extension of maternity leave [45]. The public comments on this policy focused on “childbirth security”, including children’s daily living expenses and childcare construction. From an international perspective, many high-welfare countries offer relatively long maternity and parental leave periods [11,13]. For instance, Sweden’s fertility policy promotes parental involvement in child-rearing by providing couples with up to 480 days of paid parental leave. Within this period, each parent is entitled to 90 days of nontransferable parental leave, ensuring fairness in sharing child-rearing responsibilities [46]. Public discussion on housing increased significantly from November to December 2021, following Beijing’s policy to strengthen the management of public rental housing eligibility and allocation. Moreover, the improvement of the national birth registration system received public attention for the first time during this period, and its focus was mainly on two aspects: first, public complaints about the cumbersome birth registration process. Second, this system is less popular. In light of this, China can draw lessons from the experiences of other countries regarding this policy. To alleviate the burden on families with children, in addition to cash and tax subsidies, consideration could also be given to providing housing support for families with multiple children [17]. Specific measures could include mortgage interest refunds or housing price discount policies. For example, families with two children could be offered a 50% refund of mortgage interest, while families with three children could receive a full refund. Moreover, in areas with high housing prices, policies such as a 10% discount on housing prices for families with one child, a 30% discount for families with two children, and a 50% discount for families with three children could be implemented (with the subsidy not exceeding the cap). The cost of these subsidies could be covered by increasing the supply of housing land in areas and large cities experiencing population influxes.

Since then, with the successive introduction of housing subsidies, education subsidies, childbirth subsidies, and birth registration policies by provinces and cities, public attention has become more concentrated: for example, further optimization of birth registration management methods in Guangdong Province and Henan Province and the implementation of subsidies for three-child fertility in Hanzhong city. The information was promptly disseminated to the public through online media or government organizations. The survey results of the ‘2022 China Urban Women’s Childbirth Report’ further support the above conclusions. A questionnaire survey was administered to women aged 20–45 years in first-tier, new-tier, and second-tier cities who desired to have children. The results show that the cost of raising multiple children and the decline in quality of life are the main reasons discouraging women from giving birth [47]. Additionally, regarding the policy of extending maternity leave, women who have not given birth are more concerned about salary benefits during maternity leave, pregnant women are more concerned about housing subsidies, and women who have given birth are more concerned about education resource guarantees. Combining the research conclusions of this article with the results of existing survey reports, it becomes evident that the collected text data can fully reveal subtle differences in public opinion on childbirth [48].

### Changes in sentiment and topic trends

The previous section extracted topics from online comments on the three-child supportive policy, provided insight into public focus through divided subject content, and analyzed public topics and policy attitude trends through policies released at different time points. To further analyze changes in public opinion postpolicy information release, the following sections will examine trend changes in topics and attitudes, conducting a quantitative analysis of their instantaneous or continuous impact. As a longitudinal quasiexperimental design method [49], interrupted time-series analysis (ITSA) has been used to assess the effects of community interventions [50], public policy [51], regulatory actions [52] and health technology assessment [53]. In the absence of effective controls, the ITSA performed linear regression analysis on the two periods before and after the intervention was implemented. It tested for statistically significant changes in levels at the intervention point and whether an event followed the intervention. The main advantage is controlling for a preexisting trend of an event’s decline or increase over time. ITSAs primarily rely on OLS models rather than ARIMA models due to their flexibility and wider use in interrupted time series contexts. Therefore, this study uses the ITSA to analyze changes in relevant public opinion after the release of policy information.

The standard ITSA regression model is represented in the following form:

$$Y_{t}=\beta_{0}+\beta_{1}\times time_{j}+\beta_{2}\times level_{t}+\beta_{3}\times trend_{jt}+\varepsilon_{jt}$$ (2)

where *Y*_*t*_ is the observed value of the outcome variable at each equal interval time point *t*. *time*_*j*_ is the time since the start of the study. The *level*_*t*_ is a virtual variable describing the intervention, which is 0 before the intervention and 1 for the other periods. *trend*_*j*_ is the interaction term. *β*_0_ represents the initial level of the outcome variable. *β*_1_ represents the slope or trajectory of the outcome variable before the intervention. *β*_2_ represents the instantaneous change in the outcome variable after the intervention by comparing it with the counterfactual result. *β*_3_ represents the difference between the regression slopes before and after the intervention. *ε*_*jt*_ is the random error term. This section applies the above model to this paper, and its model is:

$$sentiment_{t}=\beta_{0}+\beta_{1}\times time_{t}+\beta_{2}\times policy_{j}+\beta_{3}\times timeafterpolicy_{jt}+\varepsilon_{jt}$$ (3)

**Fig 3:**
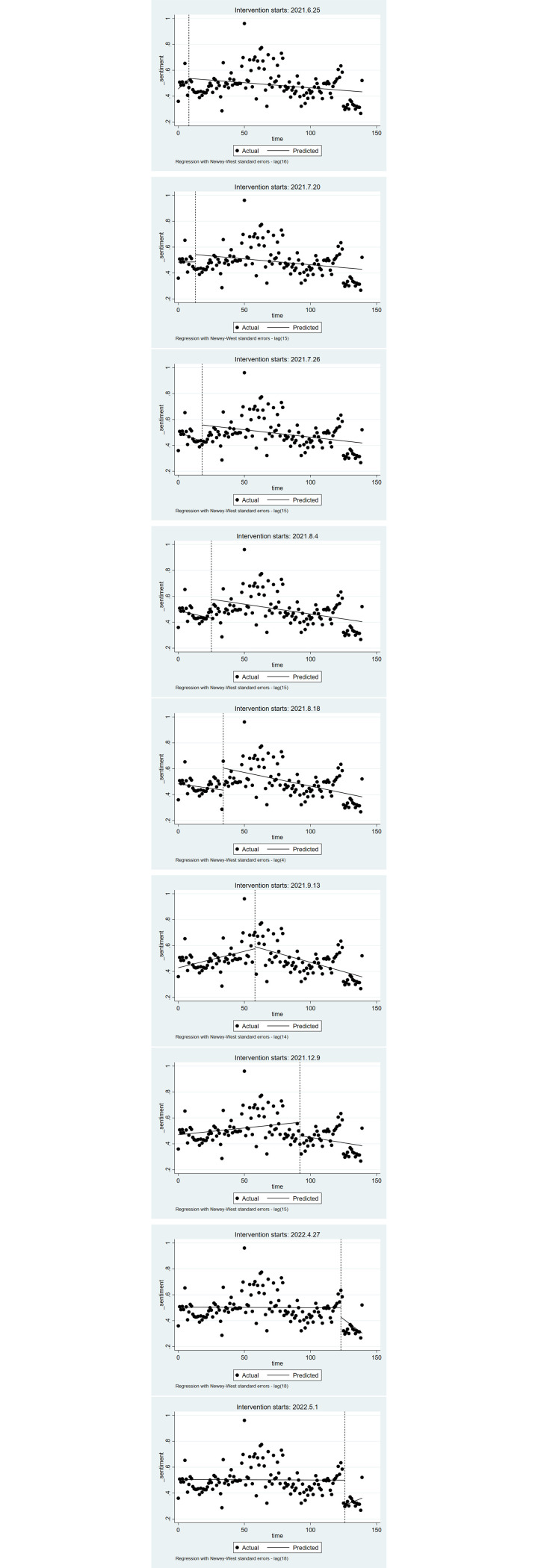
Changes in public attitudes before and after the implementation of different policies.

Based on Tables 1 and 9 and the attitude sequence values of the public, this section selects a time that corresponds to the policy release time. Additionally, the change in the trend of the attitude values and the coding percentage are relatively stable or fluctuate greatly at the selected time. In turn, this section analyzes public attitudes before and after this time. (The results are shown in Fig 3 and Table 10). The application of the interrupted time series regression model requires that the sequence does not have autocorrelation, so the Durbin-Watson method is used to check whether the sequence has 1st-order autocorrelation. The DW is between 0 and 4, and its value is close to 2 or 4, indicating that there is no autocorrelation. If the sequence has autocorrelation, this paper adopts the Newey-West method to address autocorrelation and potential heteroscedasticity. In addition, Fourier terms (sine and cosine functions) are used here to control for seasonal effects and other long-term trends.

**Table 10:** Regression parameters for different policy nodes.

No.	Policy release time	*β*_1_	*P*	*β*_2_	*P*	*β*_3_	*P*
**1**	2021.6.25	0.00096	0.150	0.0045	0.929	-0.0104	0.129
**2**	2021.7.20	0.00010	0.974	0.0567	0.225	-0.001	0.778
**3**	2021.7.26	-0.0040	0.074	0.1292	0.007	0.003	0.233
**4**	2021.8.4	-0.0024	0.075	0.147	0.008	0.00085	0.525
**5**	2021.8.18	-0.0014	0.324	0.1702	0.000	-0.0007	0.668
**6**	2021.9.13	0.00254	0.008	0.0149	0.669	-0.0054	0.000
**7**	2021.12.9	0.00105	0.110	-0.103	0.023	-0.0027	0.144
**8**	2021.4.27	-0.00005	0.907	-0.072	0.056	-0.0076	0.131
**9**	2022.5.1	-0.000035	0.929	-0.193	0.000	0.0042	0.032

Fig 3 illustrates the trend in public attitudes toward the three-child policy at key moments of its release. The subfigures show public responses on the following dates: (1) approximately 2021.6.25; (2) approximately 2021.7.20; (3) approximately 2021.7.26; (4) approximately 2021.8.4; (5) approximately 2021.8.18; (6) approximately 2021.9.13; (7) approximately 2021.12.9; (8) approximately 2022.4.27; and (9) approximately 2022.5.1.

Table 10 and Fig 3 depict the shifts in public attitudes toward various policy releases, specifically the feedback from the public on the policy agenda. Typically, the impact on public opinion following a policy release is transient, with insignificant future trends. According to the intercept items in the 3^rd^, 4^th^, and 5^th^ subfigures of Fig, on 2021.7.26 (*β*_2_ = 0.1292, *P* = 0.007), 2021.8.4 (*β*_2_ = 0.147, *P* = 0.008), and 2021.8.18 (*β*_2_ = 0.1702, *P* = 0.000), the public’s positive attitude toward policies immediately increased significantly after the policy was released. Moreover, the *β*_3_ values at these times in Table 10 are nonsignificant, which indicates that the change in slope is not significant in the long-term trend after the implementation of the policy. The content of the supportive policies of three-child maternity insurance, maternity incentives, and housing subsidies has improved. In terms of housing subsidies, the Beijing government proposed the “Notice on Strengthening Eligibility Review and Allocation Management of Public Rental Housing” on August 18, 2021. From the significance of the *β*_2_ values in Table 10, it can be seen that the two policies “Notice of the Office of the National Medical Security Bureau on the Work of Maternity Insurance in Support of the Three-Child Supportive Policy” released on 2021.7.26 and “Regulations on Population and Family Planning in Beijing” released on 2021.8.4 were quickly publicized to the public and received good feedback information, while the policy “Notice on Strengthening Eligibility Review and Allocation Management of Public Rental Housing” did not attract much public attention through the online media, which shows that the public’s timely feedback on the policies is closely related to the publicity of the media.

According to the intercept terms in the 7^th^ and 8^th^ subfigures of Fig 3, it is clear that negative public opinion surged immediately following the policy’s release on 2021.12.9 (*β*_2_ = -0.103, *P* = 0.023) and 2022.5.1 (*β*_2_ = -0.193, *P* = 0.000). However, compared to the trend before the release, the long-term trend change in the positive attitude toward the policy released in 2022.5.1 increased from the original value (as indicated by the value of *β*_3_ = 0.0042, *P* = 0.032 in Table 10), while the long-term trend change in the policy released in 2021.12.9 changed slightly (the value of *β*_3_ in Table 10 was not significant). On 2021.12.9, the National Health Commission issued the Guidelines on Improving the Birth Registration System. As seen from the public’s comments on this policy, an increase in the public’s negative attitudes does not represent dissatisfaction with the policy but rather demonstrates that the birth registration system does not affect the birth of three children. At the same time, the implementation of this policy did not have a long-term impact on public attitudes. In addition, as far as the current birth registration system is concerned, its information has not been popularized to the majority of the population, which is also one of the reasons for the public’s negative attitudes. From the slope of the 9^th^ subfigure in Fig 3 and the significant value of *β*_3_ in Table 10, the release of relevant policies in Guangdong Province on May 1, 2020, as the improvement of the birth registration management system, has a positive long-term impact on public attitudes. This is because the two types of policies are different in term of online media publicity. The topic of the national birth registration system focuses on the convenience of “online processing”, while the topic of the birth registration system in Guangdong Province focuses on “cancellation of approval for childbirth”. Compared with improving convenience, the direct cancellation of approval will have a greater impact on future three-child birth procedures, and the policy has fostered positive public sentiment and shown gradual improvement.

From the change in the slope of the 6^th^ subfigure in Fig 3 and the significance of *β*_1_ in Table 10 (*β*_1_ = 0.00254, *P* = 0.008), it is clear that the trend of positive public attitudes toward the three-child supportive policy gradually increased before the release of the policy on 2021.9.13. After the release of the policy, although there was no instantaneous change in public attitudes, the long-term trend of positive public attitudes after the implementation of the policy showed a gradual decrease (*β*_3_ = -0.0054, *P* = 0.000), which also indicates that the impact of the policy on public attitudes after its implementation was delayed. On 2021.9.13, the Guangdong Health Care Commission forwarded and implemented the Decision on Optimizing the Fertility Policy for Long-term Balanced Population Development, which covered more content. The public did not pay attention to and provide feedback on this policy. Online media reports were concentrated on highlighting the extent of “maternity leave”. According to Fig 3, the extension of maternity leave has raised women’s concerns about gender discrimination and the protection of their rights and interests in the future, and the extension of maternity leave not only affects postpregnancy employment, but also brings additional economic pressure to families, such as childbirth costs, economic income sources, and future child-rearing costs, and the public’s concerns and pressure have not been addressed in this policy. These concerns and pressures are not addressed in this policy. Hence, public negative attitudes are projected to increase over time, and such policies have not yielded positive effects on public sentiment. In addition, the public wants to increase and implement “paternity leave for men” based on maternity leave policy, as well as legal protection for women during marriage and pregnancy and fair treatment between men and women in the workplace or family life.

## Discussion

This paper organized policy text content from May 31, 2021, to June 31, 2022, crawled Weibo user comments on three-child supportive policy based on policy documents, and used sentiment analysis to capture public attitude trends before and after the release of policy information. The comment content was subject-clustered using grounded theory. Finally, an interrupted time series model was constructed to analyze the effects of public sentiment on different types of policy information. According to the above analysis, the following conclusions are drawn.

Firstly, the study highlights the public’s varying attitudes towards different aspects of the three-child policy. The public holds a positive attitude improving the improvement of policies on maternity insurance for three children, maternity incentives, and housing subsidies, but the impact on public attitudes is temporary. The public has a negative attitude toward the policy of extending maternity leave, which raises women’s concerns about future employment and the protection of marriage rights and interests. This negative attitude is persistent.

Furthermore, the analysis reveals that public feedback and attitudes towards the policy are closely related to the timing and content of online media communication. The role of the media directly affects the trend of public attitude. For instance, the ‘China Youth Netizen Social Mentality Survey Report (2022)’ conducted mixed big data analysis on 5492 netizens from various regions, age groups, and educational backgrounds. One of these conclusions pertains to the reactions of netizens when society emphasizes childbirth and proposes mandatory measures. Our research findings align with this report, indicating that when society advocates childbirth and implements mandatory measures, netizens tend to exhibit strong anti-childbirth sentiment. This underscores the importance of the media agenda in shaping public opinion, serving as a bridge between the public agenda and the policy agenda.

In the discussion about the three-child fertility policy, the most concerning content is women’s protection. This primarily focuses on three perspectives: 1) how to reduce women’s fear of childbirth before pregnancy, 2) how to improve women’s legal rights, and interests in the workplace and family after childbirth, and 3) how to pay attention to women’s physical and mental health after childbirth, such as postpartum depression, inferiority complexes, and other mental health problems. In addition to women’s protection, the public is also concerned about two issues: parenting security and economic pressure. Among them, parenting security refers to the daily living expenses of multiple children and the construction of childcare services, while economic pressure mainly comes from housing pressure after having multiple children. Research on “the current state of Chinese women’s fertility views” has been conducted in nearly 30 cities, and nearly 11,000 sample data points have been collected. The results further confirm the credibility of the conclusions of this article, namely, that the family’s economic foundation and the good physical and mental quality of both spouses are key factors in determining the desire to have children.

Lastly, the paper examines the implications of maternity security and assisted reproductive technology. The fertility willingness of infertile or single women has been mentioned frequently. These individuals express a strong desire to have children, but due to their conditions or the limitations of the medical security system, their fertility willingness cannot be satisfied. In EU countries, the proportion of children born out of wedlock averages 41.3%, while in OECD member countries, it reaches as high as 40.7%. Although China’s Civil Code stipulates that children born out of wedlock enjoy the same rights as those born in wedlock, in reality, the law provides limited support for them. When unmarried or single mothers are employed, maternity insurance does not cover related expenses, leading this group to express negative emotions and opinions on the internet. In many Western countries, single women can access assisted reproductive technologies such as egg freezing. However, in China, the development of assisted reproductive technology is somewhat restricted. For example, the ‘Human Assisted Reproduction Norms’ issued by the former Ministry of Health stipulate: ‘It is forbidden to implement human assisted reproductive technology for couples and single women who do not comply with national population and family planning laws and regulations.’ This regulation prevents single women from utilizing sperm banks, egg freezing, and other artificially assisted reproductive technologies to exercise their right to reproduction. Among the 2.1% of people in the United States who use assisted reproduction, the proportion of people in China who currently use assisted reproduction is still very small. If assisted reproductive technology is fully opened, it may reach the same proportion.

### Implications

Based on the above conclusions, this paper proposes corresponding suggestions to alleviate the current problems associated with the birth of three children. These suggestions focus on enhancing the role of media in disseminating fertility information, establishing a legal framework to protect women’s fertility, improving maternity policies, and addressing the imbalance in maternity protection systems.

First, the role of the news media is crucial in this regard. The news media integrates all parts, links, and factors of society into a whole. It provides information for public participation and becomes a bridge for communication from top to bottom. As a pivotal medium for disseminating information, popularizing knowledge, expressing public interests, and integrating and coordinating society, it directly affects the audience’s ability to discern facts through its agenda-setting function. The Danish government’s practices can serve as a reference for actively promoting fertility policies and family support measures through various media channels. The government utilizes social media platforms to disseminate information about fertility policies, family values, and parenting skills while actively engaging with the public to enhance their understanding and confidence in fertility-related matters. Additionally, the media often produces and broadcasts special reports and programs covering expert advice and introductions to government support measures, aiming to attract more attention and understanding of fertility issues. Furthermore, the government can collaborate with the media to produce family magazines and websites, providing information about fertility policies, parenting skills, and family life to help families better prepare for the arrival of newborns.

In addition to media involvement, establishing a legal framework to protect women’s fertility is crucial amid the conflict between fertility and employment, representing differing forms of productivity. This framework ensures equal employment rights and pay while minimizing fertility’s negative impact on women’s careers. In China, where high female labor participation rates are common, many couples hesitate to have children due to the challenges of childcare. To address this, the government could aim for a 50% enrollment rate of children aged 0–3 and construct at least 100,000 childcare centers. Moreover, the quality of marriage significantly influences women’s fertility. Drawing from countries such as Norway, where laws establish common marital property rights and ensure fair property division upon divorce, detailed divorce laws could protect property and rights postmarriage breakdown, ensuring fairness for both parties.

Moreover, changes in maternity policy are essential for supporting women during their transition into motherhood. Women represent an important subject of social role transformation, and their health could receive more attention, especially perinatal mental health, which is a public health field that deserves to be focused on. It is worthwhile to learn from the advanced experiences of countries such as Sweden and other European nations. For instance, establishing a comprehensive postnatal care service system, which includes postnatal medical check-ups, infant care guidance, and psychological support, can provide comprehensive assistance to new mothers. The government could also offer free or subsidized mental health services and promote the screening and treatment of postpartum depression. This ensures the early identification and intervention of problems, offering timely help and support to mothers. Furthermore, social welfare departments or community service organizations can implement family support plans, providing assistance to mothers in household chores, parenting skills training, and social support.

Lastly, addressing the imbalance in the maternity protection system in terms of group structure and urban-rural differences is vital for ensuring equitable support for all families. On the one hand, existing urban and rural basic medical insurance subsidies do not cover expenses such as maternity examinations. On the other hand, some groups such as self-employed businessmen and farmers are not involved in maternity insurance and maternity benefits. Therefore, we suggest adopting a combination of personal income tax reductions and cash subsidies. For high-income families, personal income tax can be reduced by using the number of children as a tax deduction. For low-income families, direct cash subsidies may be more practical. In this plan, we can learn from the successful cash family welfare policies implemented by some European countries. Specifically, for families with two children, a monthly cash subsidy of 1,000 yuan is proposed for each child. For families with multiple children, each child can receive a monthly cash subsidy of 2000 yuan until they reach 20 years of age. Additionally, for families with two children, a policy of halving income tax and social security contributions can be implemented, while for families with three children, full exemption from income tax and social security contributions can be provided (of course, for particularly wealthy families, a cap on subsidies can be set).

### Limitations

However, several deficiencies still exist in this paper and need further research. These include the lack of consideration of the influence of policy implementation factors, the broad categorization of public attitudes, and the reliance on qualitative data without detailed demographic differentiation. Future research should address these areas to provide a more comprehensive understanding of public attitudes towards policies.

First, in the analysis of the content of the policy text, this paper did not consider the influence of the policy implementation subject, the implementation strength, or commenters’ demographic attributes, such as geographical and age characteristics on public attitudes. These factors are crucial as they can significantly affect public perception and response to policies. Therefore, in future studies, incorporating more influencing factors will be essential to explore their impact on public attitudes comprehensively.

Furthermore, the paper quantifies the public’s attitudes toward policies only from a positive and negative perspective. This binary quantification is relatively broad and does not capture the complexity of public sentiment. In real life, positive attitudes can also be subdivided into optimism and praise, while negative attitudes can be subdivided into suspicion and disappointment. Refining the classification of public attitudes in future research will allow for a more nuanced analysis and help understand the multifaceted nature of public reactions. Additionally, this refined categorization can be used to consider the impact of multiple policy interactions on public attitudes. In light of this, outlining the regional heterogeneity in sentiment can indeed aid us in analyzing public reactions to policy releases under different regional conditions with greater accuracy. In the future, we plan to use official websites or other reliable data sources to obtain more authentic and precise user location information, thereby exploring geographical differences in sentiment analysis in greater depth.

Moreover, this study primarily relies on qualitative data, particularly textual data, which is then processed into quantitative data through text mining techniques. While this approach has its merits, it lacks detailed, differentiated processing of demographic attributes within the qualitative data. To address this limitation, future research should include in-depth interviews or questionnaire surveys targeting female groups with different statistical characteristics. This will help to overcome the limitations of qualitative data sources such as policy texts, news reports, and online comments, providing a more comprehensive understanding of public sentiment towards the three-child policy.

## Conclusions

Based on the analysis of public sentiment through Sina Weibo regarding the three-child support policy, this study identifies several key insights. It reveals significant public interest and diverse reactions to policies such as enhanced fertility insurance, incentives, and housing subsidies, which received short-term positive feedback. Conversely, policies like extended maternity leave elicited ongoing negative responses, particularly concerning women’s employment and marital rights. Central themes in social media discussions included the protection of women’s rights post-childbirth, economic pressures associated with raising multiple children, and concerns over childcare services and housing. Moreover, the study highlights strong desires for parenthood among infertile or single women, constrained by limitations in healthcare coverage. These findings underscore the complexities of public perception and suggest avenues for policymakers to refine fertility policies in response to societal needs and concerns.

## Supporting information

S1 Data (XLSX)
